# The Use of Telemedicine Access to Schools to Facilitate Expert Assessment of Children with Asthma

**DOI:** 10.1155/2008/159276

**Published:** 2007-11-19

**Authors:** David A. Bergman, Paul J. Sharek, Kathryn Ekegren, Shannon Thyne, Michelle Mayer, Mara Saunders

**Affiliations:** ^1^Department of Pediatrics, Stanford University School of Medicine, Suite 100, 770 Welch Rd, Palo Alto, CA 94301, USA; ^2^School Health Programs Department, San Francisco Unified School District, 1515 Quintara Ave, San Francisco, CA 94116, USA; ^3^Department of Pediatrics, University of California, Box SFGH, MS 6E/SFGH(nh), San Francisco, CA 94143, USA; ^4^Cecil G. Sheps Center for Health Services Research, The University of North Carolina at Chapel Hill, Chapel Hill, NC 27599, USA

## Abstract

Research has shown that access to an asthma specialist improves asthma outcomes. We hypothesized that we could improve access to expert asthma care through a telemedicine link between an asthma specialist and a school-based asthma program. We conducted a prospective cohort study in 3 urban schools to ascertain the feasibility of using an asthma-focused telemedicine solution. Each subject was seen by an asthma expert at 0, 8, and 32 weeks. The assessment and recommendations for care were sent to the primary care physician (PCP) and parents were told to contact their physician for follow-up care. Eighty three subjects participated in the study. Subjects experienced improvement (*P* < .05) in family social activities and the number of asthma attacks. Ninety four percent of subjects rated the program as good or excellent. This study demonstrates the feasibility and acceptance of a school-based asthma program using a telemedicine link to an asthma specialist.

## 1. INTRODUCTION

Asthma affects an estimated 5–10% of children at ages 0–18 and is the most common reason for childhood hospitalizations [1]. Since 1980, the national prevalence of asthma has increased to 75%, with the greatest increase occurring among children [2]. In certain high-risk inner-city locations, asthma prevalence rates exceed 20% [3]. Asthma disproportionately affects minority and low-income populations, with prevalence rates in African American and Latino children 2.5 times that of Caucasians [4, 5]. The implications of these data are substantial, as children with asthma face increased risk for behavior problems, school dysfunction, and a variety of psychosocial problems which impact their future development [6–8].

It has been 12 years since the publication of the National Asthma Education and Prevention Program guidelines for the diagnosis and treatment of asthma. Despite the widespread availability of these guidelines, there remains a persistent and significant gap between what constitutes effective asthma care for children and the actual care received, particularly for minority children and those from low-income families [9]. For example, minority and low-income children are less likely than whites and children from households with higher incomes to receive controller medications to prevent asthma exacerbations [6]. In one study of an underserved urban area, researchers found that 50% of children with asthma were not taking appropriate medications [10].

Extensive research efforts have shown that appropriate management, including the routine use of controller medications, can dramatically reduce asthma morbidity in children [11–13]. Yet, translation of these research findings into clinical practice has been slow, particularly for inner-city children from low-income families. Several strategies have been devised to increase the access to effective strategies for asthma care in underserved areas. One of these strategies is the use of school-based asthma programs to bring asthma care to a site where children are accessible on a daily basis, at least 180 days each year. This strategy dramatically diminishes patient transportation problems and scheduling difficulties. Evaluation of these programs has demonstrated increased student access to needed care and improved students’ understanding of their health and individual health outcomes; [14–16] however, the quality of care provided at these school-based clinics has been inconsistent. A study of asthma care in inner-city school-based clinics found that the adherence to the National Heart, Lung, and Blood Institute guidelines for asthma care was low among programs that did not have access to expert asthma care and a targeted asthma intervention [17]. Other studies provide evidence that asthma specialist involvement is a critical component of an effective asthma program [18–20].

Telemedicine is one potential approach to increasing access to specialty care [21]. Although the evidence base is still growing, there are identifiable positive outcomes associated with telemedicine. Telemedicine has been used in school settings to improve access to care, treat otitis media, and increase appropriate specialist referral and access to expert asthma care for patients with asthma [10, 22, 23]. Given these findings, we hypothesized that we could improve access to effective asthma care for underserved children by combining a school-based program and telemedicine to increase access to asthma specialty care.

We developed a school-based program that identified children with asthma and used a telemedicine link to elicit recommendations from an asthma specialist, which were then communicated to the patient’s primary care physician. This paper describes the feasibility and acceptance of this program.

## 2. METHODS

**Study population**

We enrolled 96 subjects from 3 elementary schools in an inner-city neighborhood in San Francisco. Potential subjects were identified through the use of school health records and/or a positive response on the International Survey of Allergy and Asthma in Children (ISAAC), which was distributed to families of children in grades K-4, indicating that they had asthma or had experienced wheezing [24]. Children between the ages of 5 and 12 who had a diagnosis of asthma on their school health record or answered positively to the ISAAC survey were considered to be eligible for study enrollment. Children who were already under the care of an allergist or pulmonologist or spoke a language other than English or Spanish were excluded from the study. This study was approved by the Stanford University School of Medicine, Institutional Research Board.

**Intervention**

Each subject participated in 4 encounters at their respective schools occurring at baseline (week 0) and weeks 8, 16, and 32. At the baseline visit, an asthma specialist assessed each subject via a telemedicine link and developed an asthma action plan and recommendations based on the patient’s classification based on NHLBI guidelines. In this setting, telemedicine involved real-time video and audio conferencing between the patient/school nurse dyad on-site at the school and the asthma specialist on site at San Francisco General Hospital. The asthma specialist directly interviewed the patient and family (if present), observed an asthma-relevant examination by the on site nurse, and reviewed spirometry data directly from the laptop computer used at the school site. At the end of this visit, each child received an asthma action plan and treatment recommendations. Study personnel sent each subject’s primary care physician a letter informing them that their patient was a participant in the study and that they would be receiving a comprehensive patient assessment with treatment recommendations. We emphasized that we would only provide recommendations and that implementation of any changes in asthma management was the responsibility of the PCP. The results of the assessment and the treatment recommendations were then communicated to the primary care physician (PCP) using a web-based program called Asthma e-Coordinator, fax, or mail, depending on their PCP’s preference. The family was then told to schedule an appointment with their PCP and take their asthma action plan and treatment recommendations to the visit.

At the week-8 visit , the asthma specialist and school nurse assessed the need for changes in therapy and communicated these recommendations to the PCP. At the week-16 visit each subject received formal, developmentally-appropriate asthma education using the American Lung Association’s “Open Airways For Schools” curriculum [25]. Children in grades K-2 had two 30-minute sessions, and subjects in grades 3–5 received four 30-minute sessions. Parents were encouraged to attend all of these education sessions. Finally, in the week-32 visit , all data collection tools were completed, and subjects graduated from the program.

**Data Collection**

At the encounters of weaks 0, 8, and 32, data collection included demographics, healthcare utilization, and assessment of parental and child asthma knowledge. The parent satisfaction survey was given at weeks 8 and 32; spirometry was conducted at baseline and week 8. One parent or guardian completed the CHSA at baseline, 8, and 32 weeks. The instrument measures functional health status and is divided into 5 domains: physical health of the child, social health of the child, social health of the family, emotional health of the child, and emotional health of the family. The test-retest reliability, internal consistency, and construct validity of this instrument have been reported previously [26, 27]. One home visit, which occurred between week-8 and week-32 visits , was made by the research nurse who used a standard checklist to assess the environment relevant to potential asthma triggers.

The CHSA also provided data on the frequency of asthma symptoms as well as utilization data including asthma-related inpatient stays, emergency department use, and unscheduled outpatient visits. The number of emergency department and unscheduled outpatient visits were summed up to produce the total number of urgent care visits. The number of urgent care visits, asthma-related inpatient stays, wheezing episodes, and asthma attacks were included in the final analysis.

Pulmonary function tests were conducted using a portable SpiroCard Spirometer (Medgraphics, St. Paul, Minn, USA) using percent predicted of normal based on height, sex, and race adjusted norms using ATS criteria [28]. Children were asked to perform a forced expiratory maneuver after maximal inhalation to measure forced expiratory volume in 1 second (FEV_1_), forced vital capacity, and forced expiratory flow in the middle half of the forced vital capacity and peak expiratory flow rate (PEFR). A total of 3 maneuvers were recorded in accordance with American Thoracic Society standards [28].

We adapted a child asthma knowledge survey from a previous study of the effectiveness of an asthma Nintendo game. The modified instrument had been pretested for acceptability [29]. The 15 item survey was administered to children of 8 years and older. Skipped items on the survey were treated as incorrect responses.

Similarly, we used a 16-item survey developed for a previous study [29] to assess parents’ evaluation of their child’s ability to manage their asthma. This survey was administered to one parent or guardian at each relevant encounter after the baseline visit. In cases where parents or guardians did not accompany the subject to the visit, efforts were made to administer the survey via phone or mail. Likewise, parental satisfaction was assessed using a previously developed 8-item survey.

**Statistical analysis**

To assess the effect of the intervention, we compared outcomes between baseline and week 8 and baseline and week 32. The outcomes of interest included health care utilization and functional health status (i.e., five domains) from the Child Health Survey for Asthma and child and parental knowledge. Because the study did not collect spirometry data at week 32, only baseline and week 8 are compared. Similarly, only week 8 and week 32 values are compared for parental satisfaction. For those subjects missing follow-up data, we used their baseline value to impute the outcome value at follow-up. Because most of our outcomes were not normally distributed, we used a nonparametric test, Wilcoxons Signed-Rank test, to perform statistical comparisons.

This research proposal was review by the institutional review boards of Stanford University School of Medicine and the San Francisco Unified School District.

## 3. RESULTS

**ISAAC survey**

ISAAC surveys were distributed to 680 students across the three schools; 310 (45%) of surveys were returned. The prevalence rate for children with self-reported asthma or wheezing on the ISAAC survey was 32%. Ninety six children with asthma were identified. Twenty four percent of the children did not have asthma indicated on their health cards and were identified only by their responses on the ISAAC survey indicating that they had experienced an episode of wheezing or whistling in the chest in the past 12 months. Eighty three (86%) of these identified asthmatic children agreed to participate in the study.

**Baseline characteristics**

Overall 54% of the study subjects were male. Seventy one percent of the subjects were African-American, 14% were Latino, and 14% were “Other.” Thirteen percent of children had Spanish as their primary language spoken at home, 78% of mothers had a high-school education or higher, and 92% of families were Medicaid eligible (see Table 1). The study population reported mild to moderate levels of disease activity based on NHLBI criteria.

**Quality of asthma care**

Twenty four percent of the study subjects did not have asthma identified on their school health card (see Table 2). Sixty nine percent of parents or guardians of children in the study were never told their children had asthma. Among children with identified asthma, 96% stated that they used inhalers and 88% used spacers; however, only 52% monitored their peak flow and 34% had asthma action plans. Sixty percent of patients had persistent asthma but only 23% of these persistent patients were taking anti-inflammatory medications. Twenty eight percent of subjects had previously seen an asthma specialist but were included in the study as they were not actively being followed by a specialist at the time of enrollment.

**Environmental control**

In the home environment, 76% of subjects had carpeting on floor of the bedroom and 47% had feathered or down pillows. Only 19% of subjects had allergy mattress and pillow covers. Thirty six percent of subjects had areas of mold or mildew in their home and 21% reported seeing cockroaches in their home.

**Study feasibility and implementation**

It took approximately 6 months to screen all eligible children, educate faculty, and initiate the telemedicine encounters. All 83 children entered in the study were seen by an asthma expert, underwent spirometry, and received an asthma action and school emergency plan within three weeks of the baseline visit. The total time commitment by the asthma specialist to see these children was 24 hours or 3.5 children/hr. Ninety eight percent of the primary care physicians agreed to participate in the study and received asthma action plans and treatment recommendations. One hundred percent of the 83 children completed the study. At the end of the study, 94% of parents rated the Asthma Telemedicine Program as excellent or good on a 5-point scale.

**Asthma outcomes**

Study subjects demonstrated a relatively high level of functioning at baseline. Scores on CHSA physical domain and the social activity of the family domain of the CHSA were in the moderately high range (80–90), and scores in the emotional health of the child and family domains and the social activity of the child domain were greater than 90. Similarly, mean spirometry results showed forced expiratory volume at 1 second (FEV_1_), forced vital capacity (FVC), FEV_1_/FVC, and forced expiratory flow (FEF_25−75_) that were all greater than 80% of predicted value. Similarly, subjects had a low level of utilization of hospital and emergency department visits as well as unscheduled visits to their primary care physician.

At the end of the intervention, subjects demonstrated significant improvement in the physical and social domains for child on the CHSA (Table 3). We were also able to demonstrate a significant improvement in child asthma knowledge and parent asthma knowledge. In addition, we observed a trend towards improvement in the number of asthma attacks in the past 2 weeks (Table 4). There were, however, no significant changes in spirometry, hospitalizations, emergency department visits, or unscheduled visits to the primary care physician.

## 4. DISCUSSION

This study demonstrated both feasibility and acceptance of a school-based asthma program that provided subspecialty access through a telemedicine link. We demonstrated that asthma specialists were able to use their time efficiently to reach a large number of children. In fact, the project rate of 3.5 children per hour was compared favorably with their experience in the asthma clinic where they were able to see only 2 children per hour (personal communication, Shannon Thyne, February 2005). We were also able to demonstrate that children can easily undergo assessment and asthma education during the school day with the assistance of a school nurse. The use of the school setting allowed for us to complete the assessments and asthma education on 100% of our participants. We were able to enlist a high degree of participation among PCPs as well as among teachers. Over 98% of the PCPs were willing to have their patients participating in the study. This may have been due to the fact that we did not “practice medicine” and only made recommendations. This mitigated any possible concerns on the part of the PCP that we may be taking their patients or fragmenting their care.

This study also demonstrated the feasibility of bringing subspecialty care to children with asthma in the context of their school. Several studies have documented improved outcomes when asthmatic children have access to subspecialty care [20, 30, 31]. By using a telemedicine link, we were able to increase the efficiency of the subspecialist’s time and were able to ensure that 100% of the study children, 92% of whom used California’s version of Medicaid, had access to subspecialty care.

Our study showed that 24% of the students who screened positive for asthma symptoms were unaware that they had asthma, which is higher than in a previous report that used more rigorous spirometry criteria for the diagnosis of asthma [32]. Our study also found that a significant proportion of students identified as having asthma were not receiving optimal treatment with a low percentage having asthma action plans, measuring peak expiratory flow rates and using spacers with their metered dose inhalers. These results are similar to other studies that have demonstrated inadequate asthma care in urban school-aged children [3, 14, 33].

Though a significant proportion of our subjects were classified as having mild and moderate persistent asthma according to the Child Health Survey of Asthma, this degree of morbidity was not reflected in the CHSA. This may be due to the fact that health status instruments reflect the degree of impact on the child and family and may not be directly related to the disease burden. Because of the relatively high scores on the preintervention subscales of the CHSA, there was less opportunity to demonstrate a more significant impact. Previous work however has demonstrated that the CHSA is the most stable asthma outcome measure and best demonstrates improvement over time [34]. Compared to baseline, health postintervention outcomes were improved on two of the 5 subscales of the CHSA.

We did not find evidence of an effect on healthcare utilization. There are several possible reasons for a lack of effect of the intervention on visits to the emergency department or the physician’s office. First, the number of subjects in the study may have been too small to detect a significant difference in relatively uncommon events. Second, the intervention did not ensure that the study subjects made a visit to their PCP to have their medical regimen adjusted in light of recommendations from the telemedicine encounter. In fact, many of our families did not know the name or location of their PCP. This lack of an effective partnership between our study families and their PCP made it difficult to leverage expert recommendations and asthma action plans and ultimately decrease visits to the emergency department and the physician office. Lastly, the intervention for many of our children began in the spring and with final outcomes assessed during the fall and winter. A lack of impact on utilization may have been secondary to the increased incidence of respiratory infections during the fall and winter months leading to increased symptom burden and utilization of health care services.

This study has several important limitations. Without a control group, it is difficult to know whether or not our results may be secondary to secular factors such as seasonality that are unrelated to the intervention. Second, we did not employ the “gold standard” for measuring asthma outcomes, the number of days with asthma symptoms. Our previous work in this community had raised serious doubts about the reliability of using diary data to measure symptom days [29]. It may be that the two-week recall window used on the CHSA was insufficiently sensitive to detect improvement in asthma outcomes. Third, the inclusion of relatively low morbidity asthmatics, with low levels of utilization, relatively high-pulmonary function, and high functional health status gave little opportunity for statistically significant improvement with our intervention. The study was underpowered to show significant improvement in these mildly effected outcomes, but did suggest several important trends in the positive direction.

Another limitation of this study was our inability to monitor and assess changes in the care received from the primary care providers. In an effort to maintain an appropriate link to the primary care providers of our subjects, we decided to send the asthma specialist recommendations to them for implementation rather than implementing these recommendations ourselves. This model values the continuity and relationship between primary care provider and subject, but did not ensure that the asthma specialist recommendations were implemented. Hence, the intervention tested was actually the access to subspecialist recommendations rather than implementation of these recommendations themselves. In their study of a school-based program that prompted pediatric primary care physicians as to appropriate asthma care, Halterman and colleagues found poor compliance on the part of the primary care physicians with the recommended care [35]. Thus, we suspect that inconsistent implementation of the subspecialist recommendations by the primary care provider negatively effected our outcomes.

## 5. CONCLUSION

This study has demonstrated the feasibility, efficiency, and acceptance of using a telemedicine link to bring expert asthma care to underserved children in the school setting. The results also demonstrate the effectiveness of the program in ensuring that children identified with asthma receive a comprehensive assessment, asthma action plan, and asthma education. The use of a telemedicine link also allowed for a more efficient use of the asthma subspecialist’s time when contrasted with hospital-based asthma clinics. Finally, there were significant improvements in functional health status outcomes. While the results suggest a positive impact on asthma outcomes, a true assessment of program impact will require a randomized, controlled trial.

## Figures and Tables

**Table 1:** Study subject characteristics.

		Number	%
Child's sex	Male	48	54.5%
Child's race	African-american	62	70.5%
	Latino	13	14.8%
	Other races	13	14.8%
Transportation to medical care	Car	61	75.3%
	Other	18	22.2%
Marital status	Respondent^*^ married	29	44.6%
	Respondent separated or divorced or widowed	7	10.8%
	Respondent single	39	60.0%
Home language	English primary language at home	74	87.1%
Mother's education	< 12 years	16	21.3%
	12 or equivalent	31	41.3%
	> 12 years	28	37.3%
Caregiver/employment	Primary caregiver employed	54	61.4%
	Other caregiver employed	32	36.4%
Insurance source	Medical	50	92.6%
	Other	4	7.4%

**Table 2:** Quality of care and patient education.

	Number of nonmissing observations	Number with affirmative response	% with affirmative response
Ever told child has asthma	87	60	69.0%
Ever saw an asthma specialist	87	25	28.7%
*For children identified with asthma*
Child ever taught to use an inhaler	85	62	72.9%
Child ever taught to use a spacer	86	53	61.6%
Child ever taught to take a peak flow	84	33	39.3%
Child ever taught to take about peak flow zones	85	24	28.2%
Child ever given care plan by MD/RN	86	39	45.3%
Child ever taught to control asthma triggers	85	48	56.5%
Child now taking anti-inflammatory meds	86	20	23.3%

**Table 3:** Functional health outcomes child health survey of asthma.

	Mean baseline	Mean week 8	*P*-value baseline versus week 8	Mean week 32	*P*-value baseline versus week 832
Physical	84.2	84.4	NS	87.4	.009
Social activity-child	92.4	92.0	NS	94.7	.008
Social activity-family	92.2	93.7	NS	95.2	NS
Emotional health-child	91.8	90.7	NS	91.5	NS
Emotional health -family	80.1	78.9	NS	81.1	NS

Statistical comparisons performed using Wilcoxon Signed-Rank test.

**Table 4:** Asthma outcomes.

	Mean baseline	Mean week 8	*P*-value baseline versus week 8	Mean week 32	*P*-value baseline versus week 32^*^
**Knowledge and satisfaction**
Child knowledge	16.6	17.7	.007	17.4	0.03
Parent knowledge	11.9	13.8	< .001	14.0	< .001
Parent satisfaction	—	.85	—	.87	0.02
**Asthma symptoms and utilization**
# wheezing episodes, past 2 weeks	1.18	1.44	NS	0.99	NS
# asthma attacks, past 2 weeks	0.33	0.58	NS	0.153	(.07)
# overnight in hospital, past 2 weeks	0.012	0.036	NS	0.012	NS
# ED visits, past 2 weeks	0.059	0.082	NS	0.024	NS
# sick visits, past 2 weeks	0.072	0.24	.05	0.072	NS
**Spirometry**
FEV1	96.5	96.7	NS	—	—
FEF2575	86.9	86.3	NS	—	—
FEF Max	97.5	98.0	NS	—	—
FEV/FVC	94.5	95.9	NS	—	—

^*^For parent satisfaction, the comparison involves week 8 and week 32.
